# Good agreement of conventional and gel-based direct agglutination test in immune-mediated haemolytic anaemia

**DOI:** 10.1186/1751-0147-54-10

**Published:** 2012-02-08

**Authors:** Christine J Piek, Erik Teske, Martin W van Leeuwen, Michael J Day

**Affiliations:** 1 Department of Clinical Sciences of Companion Animals, Utrecht, Utrecht University, PO Box 80154, 3508 TD Utrecht, The Netherlands; 2 School of Veterinary Sciences, University of Bristol, Langford BS40 5DU, UK

**Keywords:** Haemolysis, Anaemia, Diagnostic test validation, Laboratory

## Abstract

**Background:**

The aim of this study was to compare a gel-based test with the traditional direct agglutination test (DAT) for the diagnosis of immune-mediated haemolytic anaemia (IMHA).

**Methods:**

Canine (n = 247) and feline (n = 74) blood samples were submitted for DAT testing to two laboratories. A subset of canine samples was categorized as having idiopathic IMHA, secondary IMHA, or no IMHA.

**Results:**

The kappa values for agreement between the tests were in one laboratory 0.86 for canine and 0.58 for feline samples, and in the other 0.48 for canine samples. The lower agreement in the second laboratory was caused by a high number of positive canine DATs for which the gel test was negative. This group included significantly more dogs with secondary IMHA.

**Conclusions:**

The gel test might be used as a screening test for idiopathic IMHA and is less often positive in secondary IMHA than the DAT.

## Background

Immune-mediated haemolytic anaemia (IMHA) is caused by the binding of antibodies to the surface of red blood cells (RBCs). The production of such antibodies can be a primary autoimmune phenomenon or be associated with underlying neoplasia, chronic infections, inflammatory disease or be triggered by exposure to drugs or vaccines (secondary IMHA) [1]. The criteria used to define IMHA in dogs and cats vary between different studies; however, it is generally accepted that a positive direct agglutination test (DAT), marked spherocytosis or true autoagglutination are three hallmarks of canine IMHA. At least one of these changes must be present in a patient with haemolytic anaemia to warrant a diagnosis of IMHA [2].

The DAT demonstrates the presence of anti-erythrocyte antibodies by incubating a suspension of washed patient erythrocytes with polyvalent or monovalent antisera specific for immunoglobulin or complement. More recently, a gel-based test has been developed (Diamed, Cressier, Switzerland) [3]. The gel test is fast and easy to perform and has the potential for in-house use. A whole-blood sample may be used (instead of washed and resuspended RBCs) and the test uses a smaller blood volume than that required for the DAT [4].

The aims of this study were: (1) to perform a comparison between the feline and canine gel test with the traditional DAT in a two-centre study, and (2) to assess the usefulness of the gel test as a diagnostic tool in IMHA in dogs. After completion of this investigation the authors were notified by the manufacturers that they were withdrawing this particular gel test from the market. However, we believe it worthwhile to publish the results of the study as it is possible that similar diagnostics based on this immunological principle may become available in the future and help in standardizing anti-erythrocyte antibody testing between laboratories.

## Methods

### Study design and definition of sample material and patients

The comparison of the gel test with the DAT was performed on samples from referral patients and samples sent by private practitioners for DAT testing submitted to two centres, the Utrecht University Veterinary Diagnostic Laboratory (UVDL) and the Bristol Clinical Immunology Diagnostic laboratory (BCIDL).

In the UVDL, 126 canine samples with a haematocrit (Ht) below the lowest end of the reference range (0.42 l/l) were included in the study (October 2008 - July 2009). In the BCIDL, 74 feline (September 2007 - October 2009) and 121 canine samples (July 2007 - August 2008) were included in the study without restriction on the Ht. At the UVDL the experiments were approved by the responsible ethical committee as required under Dutch legislation. At the BCIDL all residual blood samples for gel testing were used with permission of the animal owner and in accordance with UK legislation.

Samples coming from private practitioners were sent in by mail overnight. Both the gel test and the DAT were performed within 24 hrs of receipt of the samples. Until then samples were kept at 4°C.

The assessment of the usefulness of the gel test as a diagnostic tool for IMHA was performed on cases from the UVDL only. For this, all dogs that had either a positive gel test or DAT were categorized clinically as having: (1) idiopathic IMHA, (2) secondary IMHA, or (3) anaemia that was not immune-mediated (no IMHA).

The inclusion criteria for idiopathic IMHA were: (1) acute onset anaemia due to haemolysis, (2) Ht below 0.35 l/l, (3) a positive DAT or spherocytosis. In cases where an underlying disorder or recognized trigger factor (e.g. infection, neoplasia or administration of drugs) was identified the dog was classified as having secondary IMHA. In order to make this classification, information available at the time of presentation as well as follow-up and post-mortem results were reviewed.

### Laboratory tests

#### Gel test

The gel test (Diamed, Cressier, Switzerland) was performed according to the manufacturers' instructions. The test is presented in the form of a small plastic card within which there are six columnar tubes with an overlying reservoir of wider diameter. The tube contains a gel matrix impregnated with rabbit polyvalent DAT-reagent specific for either dog or cat. The cards used by the two centres were the same, but the UVDL was also supplied with separate negative control cards that comprised tubes containing only the gel matrix without antibody. Using the diluent supplied by the manufacturer a 0.8% RBC suspension was prepared and 50 μl of this suspension was pipetted into the reservoir above the gel. In the BCIDL the patient RBC were first washed twice in phosphate buffered saline (PBS; pH 7.4, 0.01 M) and then resuspended in the diluent. The card was centrifuged in a purpose-designed centrifuge supplied by the manufacturer that runs at a set centrifugal force for a defined time period. Following centrifugation, the tubes were examined to determine the distribution of the red cells. In a negative test the cells were pelleted at the bottom of the tube. Where cells were distributed within the cell matrix the test result was recorded as weak positive. Where cells were retained at the top of gel the test was reported as positive.

#### DAT

In both laboratories a similar DAT protocol using was used as described previously [5,6]. Erythrocytes from each dog were washed three times in PBS and resuspended at 5% in PBS. All reagents were titrated in PBS across the rows of a microtitre tray. An equal volume of the 5% erythrocyte suspension was added to each well and to control wells containing PBS alone. The microtitre trays were incubated until erythrocytes in the control wells had settled. Agglutination was assessed visually, and the titre was recorded as the reciprocal of the last dilution of each antiserum giving a positive agglutination reaction. The DAT was considered positive if at least one of the reagents resulted in a positive titre and the control wells showed no agglutination.

##### UVDL

The test was performed with monovalent canine Coombs' reagents: rabbit anti-dog IgG (Fc), anti-dog IgG (H+ L), and anti-dog IgM (Fc) antibodies (Nordic Laboratories, the Netherlands). Tests with anti-IgG reagents were performed at 37°C and tests with anti-IgM at 4°C. A titre ≥ 16 was considered positive with any antibody.

##### BCIDL

The test was performed using a polyvalent canine Coombs' reagent (raised in rabbit) with specificity for canine IgG, IgM and complement C3 (ICN Pharmaceuticals, United Kingdom) and monovalent rabbit anti-dog IgG (Fc), rabbit anti-dog IgM (Fc) and goat anti-dog C3 (Nordic Laboratories). All reagents were pre-absorbed against a pool of normal canine erythrocytes. The test was performed in duplicate plates, one of which was incubated at 4°C and the other at 37°C. A titre ≥ 20 was considered positive.

The feline DAT was performed at both temperatures with three species-specific antisera: polyvalent feline Coombs' reagent (ICN Pharmaceuticals, United Kingdom), rabbit anti-cat IgG (Fc) and rabbit anti-cat IgM (Fc) (Nordic Laboratories) [7].

#### Haematology

##### UVDL

The ADVIA 120 (Siemens Medical Solutions, Diagnostics, USA) was used to determine Ht, total white blood cell count (WBC), differential WBC counts, platelet count and reticulocyte count. All blood smears were examined for the presence of spherocytes at the time of this study by MWvL.

##### BCIDL

Haematology results were available for samples that had been submitted for concurrent haematological examination and DAT testing. A Cell Dyn 3700 analyzer (Abbot, USA) was used to determine Ht, WBC, differential WBC count, platelet and reticulocyte counts. These analyses were performed by the Langford Veterinary Services Diagnostic Laboratories.

### Statistics

The results of the gel test were compared with the DAT by plotting the log titres of the DAT against the gel test results and determining correlation by ANOVA. Since in the UVDL only samples with a Ht below the reference range were tested, we created for comparative purposes a subset of 48 canine BCIDL samples with Ht below the reference range used by the BCIDL (0.35 - 0.55 l/l). The percentage agreement between the gel test and the DAT beyond chance, kappa, was calculated with Win Episcope 2 http://www.clive.ed.ac.uk/winepiscope. To test whether the kappa was significantly different from 0, a one sided Z-test was used.

A comparison of the number of gel test and DAT outcomes, pattern of reactivity, and the titre in the DAT for dogs in the three clinical categories was made with Fisher's exact test. All statistical analyses were performed in R http://www.r-project.org. P < 0.05 was considered significant.

## Results

### Samples

Thirty two of the 126 UVDL dogs were classified as having idiopathic IMHA, 31 as secondary IMHA, and 63 as no IMHA. The underlying disorders in the dogs with secondary IMHA included neoplastic disease (six haemopoietic tumours, one cutaneous mast cell tumour and two abdominal masses), parasitic infections (one *Babesia canis *and one *Ehrlichia canis *), bacterial infections (two urogenital tract infections, two cases of bacterial lymphadenitis), immune-mediated disorders (two idiopathic inflammatory bowel disease, one immune-mediated thrombocytopenia, one systemic lupus erythematosus, four pure red cell aplasia and three bone marrow dysplasia), central neurological disease (two dogs), and in three dogs no definitive diagnosis was made.

Of the 32 dogs classified as having idiopathic IMHA, 20 animals were still alive at least 6 months after the last sample was entered into the study. Eleven dogs died due to IMHA within the first 2 weeks after diagnosis. In these dogs extensive diagnostic testing did not reveal an underlying disorder, nor did the post-mortem examination performed in two of these cases. All but six of the dogs with idiopathic IMHA had a regenerative anaemia at the time of diagnosis (corrected reticulocyte count ≥ 1.5%; range 0.4 - 9.6%); one of these dogs died but the other five developed a regenerative anaemia during the first week of treatment.

### Performance of the Gel Test versus the DAT

The log titres of the UVDL DAT were significantly associated with a negative result in the gel test, versus the combined weak positive and positive gel test results. For the BCIDL data there was a significant association between the log titres of the DAT and all three outcomes of the gel test (data not shown). Kappa statistics were performed using both the DAT and gel test results as binary data. The comparative analysis of the two test methods for canine samples for the two laboratories is summarized in Table 1.

**Table 1:** Results of Gel test versus the DAT as performed in the UVDL and BCIDL.

Gel test	UVDL canine DAT			BCIDL canine DAT			BCIDL feline DAT			BCIDL Canine DAT Ht < 0.35 l/l		
	**-**	**+**	**Total**	**-**	**+**	**Total**	**-**	**+**	**Total**	**-**	**+**	**Total**

Negative	52	26	78	73	0	73	46	11	57	36	0	36
Positive	7	41	48	8	40	48	2	15	17	1	11	12

Total	59	67	126	81	40	121	48	26	74	37	11	48

Kappa	0.48*			0.86*			0.58*			0.94*		

P < 0.0001; Tested against H_0 _that all observed agreement is based on chance

### Pattern of Isotype Reactivity in DAT versus the Gel Test Outcome

**Figure 1:**
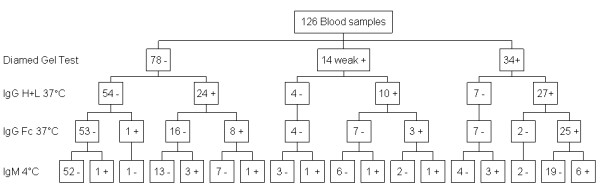
**DAT antibody isotype reactivity pattern for 126 canine blood samples tested in the UVDL**. Coombs' test antibody isotype reactivity pattern for 126 canine blood samples tested in the UVDL.

**Figure 2:**
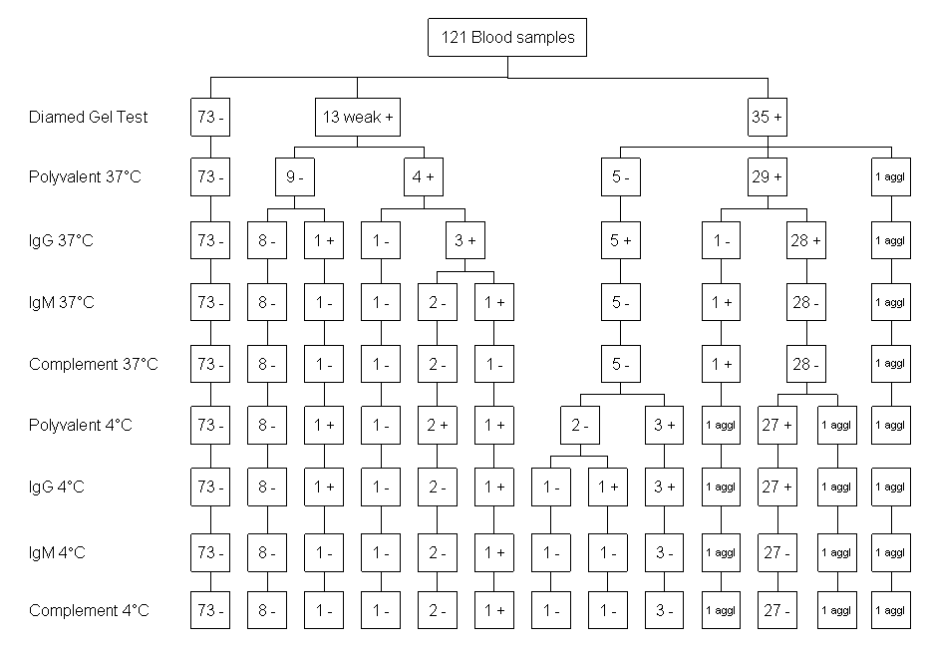
**DAT antibody isotype reactivity pattern for 121 canine blood samples tested in the BCIDL**.

**Figure 3:**
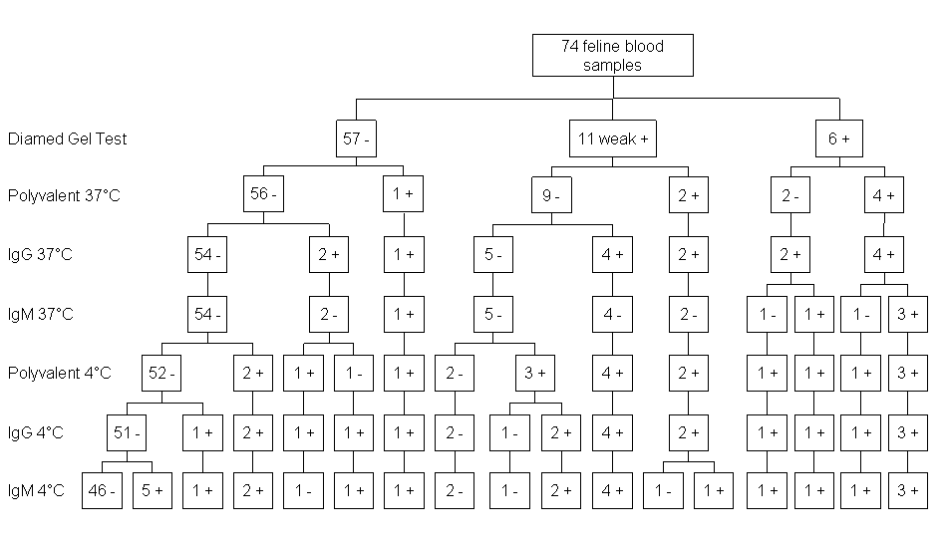
**DAT antibody isotype reactivity pattern for 74 feline blood samples tested in the BCIDL**.

The pattern of antibody reactivity in the DAT in relation to the gel test outcome is shown in Figures 1, 2 and 3.

### Relation with Clinical Classification

**Figure 4:**
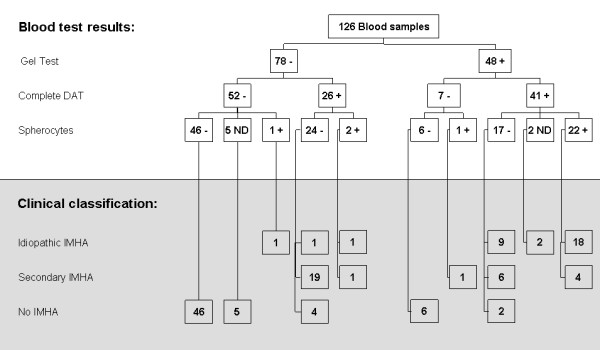
**Gel Test, DAT and spherocytosis in dogs with idiopathic IMHA, secondary IMHA and no IMHA**.

The difference in distribution of the clinical outcome over the DAT and gel test results was significantly different (P < 0.0001) (Figure 4). Of the 52 dogs negative for both the gel test and the DAT, 51/52 (98%) did not have IMHA. Only one of these dogs (2%) had idiopathic IMHA. The gel test and the DAT in this dog were performed one day after the start of immunosuppressive treatment.

A prozone phenomenon was noted in 17 of the 126 UVDL canine samples. In all but one of these 17 samples the DAT titre was positive. In 5 of the 17 dogs with prozones, the titre of the prozone was ≥ 16 (the cut-off titre at which the UVDL DAT is regarded as positive). Three of these five dogs had idiopathic IMHA and two had secondary IMHA. Of the 17 dogs with prozone effects, nine had idiopathic IMHA and the other eight had secondary IMHA.

The number of DAT reaction patterns in the dogs with idiopathic IMHA was significantly different from dogs with secondary IMHA (P < 0.0001) and no IMHA (P < 0.0001), and also for the comparison of dogs with secondary IMHA versus dogs without IMHA (P < 0.0001) (Table 2). Samples from dogs with idiopathic IMHA more often achieved higher titres than those from dogs with secondary IMHA, but this was not statistically significant (P = 0.34) (Table 3).

**Table 2:** UVDL DAT reactivity patterns in dogs having idiopathic IMHA, secondary IMHA, and no IMHA.

IgG H+L 37°C	IgG Fc 37°C	IgM 4°C	Idiopathic IMHA	Secondary IMHA	No IMHA
+	+	-	20	7	1
+	-	-	4	12	5
+	-	+	0	4	0
+	+	+	4	4	0
-	+	-	0	1	0
-	+	+	0	0	0
-	-	+	3	2	0
-	-	-	1	1	57
			
		Total	32	31	63

**Table 3:** Number of dogs for each titre reached in the UVDL complete DAT for dogs having idiopathic IMHA, secondary IMHA, and no IMHA.

	Highest titre reached in the complete DAT														
	0	2	4	8	16	32	64	92	128	256	512	1024	2048	4096	Total
Idiopathic IMHA	1	0	0	0	5	3	2	0	4	7	1	0	3	6	32
Secondary IMHA	0	0	1	0	7	4	6	1	3	2	1	2	2	3	31
No IMHA	44	8	0	5	1	2	1	0	1	0	0	1	0	0	63
Total	45	8	1	5	13	9	9	1	8	9	2	3	5	9	126

## Discussion

The DAT is an accepted method of detection of anti-erythrocyte antibodies and an essential part of the diagnosis of IMHA [2]. The first aim of this study was to compare the results obtained in the DAT versus the gel test. The high number of samples, 247 canine and 74 feline samples, creates a relatively powerful study, as for such comparative investigations 40 samples has been described as sufficient [8]. Analysis of the correlation between the log titres of the DAT results and the ordinal gel test results revealed that the latter can be interpreted as binary test results. Therefore kappa statistics were chosen as the appropriate method to compare the two tests in the absence of a "gold standard" for the diagnosis of IMHA [8,9]. The kappa values for the comparison of the canine BCIDL (0.86) as well as the kappa of 0.94 for the BCIDL canine samples with Ht below 0.35% can be interpreted as "almost perfect agreement" and the kappa for the feline BCIDL samples (0.58) and kappa for the UVLD canine samples (0.48) as "moderate agreement" using the classification of Landis and Koch [9].

The use of an extended DAT protocol such as performed by both laboratories in this study in their diagnostic support for a university referral hospital has been questioned for samples derived from first opinion clinical practice [10]. A still unresolved issue is the use of multiple dilutions of antisera to prevent false negative DAT outcomes due to the prozone effect [5,11]. Five of 17 UVDL samples had prozones with a titre around the cut-off value defined for the DAT and would have been missed if higher dilutions of antiserum had not been used.

A second issue concerns the inclusion of multiple monovalent reagents to increase the sensitivity of the DAT [5,12,13]. Indeed in the BCIDL canine samples, six positive DATs would have been missed if the test had relied on polyvalent antisera only. This finding corroborates a previous study by this laboratory in which 11 of 77 dogs with IMHA would have had a negative DAT had the extended test protocol not been used [5].

The third issue related to the DAT is the clinical relevance of performing an extended DAT with full determination of the pattern of antibody reactivity instead of use of a polyvalent reagent only, such as in the gel test. In human IMHA the antibody reactivity pattern is of diagnostic relevance [14]. In dogs, however, the clinical relevance remains debated. C3b and IgM reactivity have been reported with higher frequency in secondary IMHA [5,6] and IgM reactivity at 4°C was also more often seen in the dogs with secondary IMHA in the present study (Table 2). The clinical significance of cold agglutinating antibodies is also widely debated. There is a belief that they are less significant as they are rarely active at body temperature [2], but few studies have investigated the complete temperature gradient of the reactivity of these immunoglobulins.

Despite the fact that the DAT is an accepted test for the diagnosis of IMHA [2], it is by no means internationally standardized. To realise such standardisation between laboratories will be very difficult, if not impossible. A commercially produced test, such as the gel test, might be better suited to this task. Therefore, in addition to the comparative study of the two diagnostic procedures, the second goal of the present study was to investigate the value of the gel test as a diagnostic tool for IMHA. In the absence of a gold standard we defined clinical categories of idiopathic IMHA, secondary IMHA and no IMHA on the basis of a combination of diagnostic criteria present at clinical presentation and as used by others [2,15-20]. Additionally, these case definitions included follow-up and pathology data in order to prevent misclassification. It can be argued that this clinical classification is based on subjective criteria; however, until better methods have been found, it is consistent with current clinical practice.

The gel test identified fewer positive samples than the DAT despite the fact that in essence it is also a test reliant on the use of a polyvalent antiserum (Figure 4). The positive DAT samples that were missed by the gel test (n = 27) were mainly from dogs with secondary IMHA (n = 20). Only three cases of idiopathic IMHA were missed. Since the gel test is faster and more easily performed than the conventional DAT it might be used as a screening test for IMHA, but if negative, IMHA cannot be excluded and an extended DAT should be performed. This observation contrasts, however, with the findings in human samples where the micro-column gel test is found to be more sensitive than the DAT [21,22].

## Conclusions

Overall, in the present study there was a good agreement between the results of the DAT and the gel test. Additionally, the gel test performed well in identifying dogs with idiopathic IMHA, but missed most of the samples that were from dogs with secondary IMHA. From a laboratory viewpoint these samples also differed in having lower titres in the DAT, more frequent IgM positivity and almost no spherocytosis.

The gel test offers an appealing option as an initial screening test for idiopathic IMHA and a much-needed means of standardizing anti-erythrocyte antibody testing. It is therefore unfortunate that the current test has recently been withdrawn from the market. It is entirely feasible that the same or a similar diagnostic system may be reintroduced in the future as often occurs in the veterinary marketplace. The results of the present study help validate a specific product, but equally may be used to support the development and marketing of alternative products based on the same immunological principle in the future.

## Abbreviations

(DAT): Direct Agglutionation Test; (IMHA): immune-mediated haemolytic anaemia; (RBC's): Red Blood Cells; (UVDL): Utrecht University Veterinary Diagnostic Laboratory; (BCIDL): Bristol Clinical Immunology Diagnostic laboratory; (PBS): Phosphate Buffered Saline; (WBC): White Blood Cell Count.

## Competing interests

The gel kits used in this study were kindly provided by Diamed Benelux BV.

## Authors' contributions

ML carried out the gel tests and the DATs in the UVDL. CP carried out the statistical analysis. CP, MD, and ET participated in the design of the study and helped to draft the manuscript. All authors read and approved the final manuscript.
